# Quantifying team cooperation through intrinsic multi-scale measures: respiratory and cardiac synchronization in choir singers and surgical teams

**DOI:** 10.1098/rsos.170853

**Published:** 2017-11-06

**Authors:** Apit Hemakom, Katarzyna Powezka, Valentin Goverdovsky, Usman Jaffer, Danilo P. Mandic

**Affiliations:** 1 Department of Electrical and Electronic Engineering, Imperial College London, London SW7 2AZ, UK; 2 Department of Vascular Surgery, Imperial College London, London SW7 2AZ, UK

**Keywords:** multivariate empirical mode decomposition, multivariate synchrosqueezing transform, intrinsic multi-scale analysis, coherence, respiration, heart rate variability

## Abstract

A highly localized data-association measure, termed intrinsic synchrosqueezing transform (ISC), is proposed for the analysis of coupled nonlinear and non-stationary multivariate signals. This is achieved based on a combination of noise-assisted multivariate empirical mode decomposition and short-time Fourier transform-based univariate and multivariate synchrosqueezing transforms. It is shown that the ISC outperforms six other combinations of algorithms in estimating degrees of synchrony in synthetic linear and nonlinear bivariate signals. Its advantage is further illustrated in the precise identification of the synchronized respiratory and heart rate variability frequencies among a subset of bass singers of a professional choir, where it distinctly exhibits better performance than the continuous wavelet transform-based ISC. We also introduce an extension to the intrinsic phase synchrony (IPS) measure, referred to as nested intrinsic phase synchrony (N-IPS), for the empirical quantification of physically meaningful and straightforward-to-interpret trends in phase synchrony. The N-IPS is employed to reveal physically meaningful variations in the levels of cooperation in choir singing and performing a surgical procedure. Both the proposed techniques successfully reveal degrees of synchronization of the physiological signals in two different aspects: (i) precise localization of synchrony in time and frequency (ISC), and (ii) large-scale analysis for the empirical quantification of physically meaningful trends in synchrony (N-IPS).

## 1. Introduction

Cooperative human activities require high degrees of mental and physical synchronization among multiple participants, to the extent that synchrony underpins performance level in activities, such as choir singing, playing music in an ensemble, rowing, flying an aeroplane with a co-pilot or performing surgical procedures. When it comes to quantifying the degree of synchronization among participants, synchrony in physiological responses has been reported in respiration and heart rate variability (HRV) among the choir members [1,2].

Synchrony in respiration among the choral singers is a result of their breathing rhythm being dictated by the tempo and demands of a musical score; that is, they typically perform the short inhalation and long exhalation in unison. In addition to the voluntarily controlled breathing, the respiration is also involuntarily controlled by the autonomic nervous system, which comprises the sympathetic (SNS) and parasympathetic (PNS) nervous subsystems. The SNS also accelerates other functions, such as the arterial blood pressure and heart rate [3–5], by dilating bronchioles in the lungs, and by regulating neuronal and hormonal responses to stimulate the body. The PNS, on the other hand, slows down physiological functions when the body is at rest.

The interplay between the SNS and the PNS, among other factors, manifests itself in variations of the timing of the cardiac cycle—HRV—in response to both external and internal factors. Changes in HRV are commonly evaluated in two frequency bands: (i) the low-frequency (LF) band, 0.04–0.15 Hz, which is linked to the interaction of the SNS and PNS, and (ii) the high-frequency (HF) band, 0.15–0.4 Hz, which primarily reflects the activity of the PNS [6,7]. In addition, it is well understood that breathing modulates HRV via a phenomenon referred to as the respiratory sinus arrhythmia (RSA), whereby the heart rate accelerates during inspiration and decelerates during expiration. The RSA is usually attributed to the activity of the PNS, so that the HF component of HRV is dominated by the changes in heart rate induced by breathing.

In an attempt to quantify the degrees of synchronization in the singers’ physiological responses (respiration and HRV), a quantitative measure of the level of cooperation has been recently proposed in [1]. This is achieved via the assessment of phase relationship between multiple physiological responses, based on the intrinsic phase synchrony (IPS) and intrinsic coherence (ICoh) measures proposed in our recent work [1,8] under a framework referred to as *intrinsic multi-scale analysis*. The algorithms under this framework are capable of quantifying intra- and inter-component dependence of a complex system, such as multiple synchronies and causalities. The IPS and ICoh are implemented through a combination of the novel data-driven multivariate signal decomposition algorithm called *noise-assisted multivariate empirical mode decomposition* (NA-MEMD) (see [9,10] and appendix A.1 for more details) and two standard data-association measures, phase synchrony and coherence. As desired, the IPS accounts only for phase information between dependent signals, regardless of the differences in the magnitude of intrinsic oscillations between data channels obtained using NA-MEMD. The time and frequency aspects of synchrony, however, are not highly localized using this algorithm. Conversely, using ICoh, the degree of signal dependence can be quantified as a function of frequency, at a loss of the time dimension, because ICoh is computed over the whole dataset.

Despite several limitations, when used in conjunction with IPS and ICoh, NA-MEMD is an efficient multichannel data processing method. It is an extension of the empirical mode decomposition (EMD) [11,12] to multivariate cases, with the assistance of noise in order to enforce the dyadic filterbank property. The EMD is essentially an adaptive, data-driven method for the analysis of nonlinear and non-stationary univariate time series. It employs the so-called sifting process to decompose a given signal into its multiple physically meaningful narrow-band amplitude/frequency modulated (AM/FM) components, which are referred to as intrinsic mode functions (IMFs) and are used as bases for signal representation.

Unlike conventional projection-based time–frequency (TF) algorithms, such as the short-time Fourier transform (STFT) and the discrete wavelet transform (DWT), the IMFs—the adaptive basis functions within EMD—enable physical interpretation for nonlinear signals, because IMFs are theoretically designated as intrinsic oscillations with intrawave amplitude and frequency modulation, with a physical meaning that the amplitudes and frequencies of these basis functions can *intrinsically and nonlinearly* vary over time. Standard algorithms, on the other hand, employ rigid basis functions with fixed frequencies (cosine in STFT, wavelet function in DWT), and therefore *linear* superpositions of additional harmonic components are required to represent nonlinear signals, thus spreading energy over a wider, higher frequency range and not admitting physical interpretation. Applications of EMD range from biosignal analysis [13,14], through to mechanical systems [15] and seismology [16].

Owing to the empirical nature of EMD, its direct component-wise application to multivariate signals may result in: (i) IMFs with different intrinsic oscillatory components (modes) across multiple data channels for a given IMF index—a phenomenon known as *mode mixing*, and (ii) multiple IMFs containing similar oscillatory modes for a given data channel—a phenomenon referred to as *mode splitting*. To mitigate these problems in multivariate scenarios, several extensions to EMD have been proposed, which include the complex EMD [17], rotation-invariant complex EMD [18], bivariate EMD [19], trivariate EMD [20], multivariate EMD (MEMD) [10,21] and noise-assisted MEMD (NA-MEMD) [9]. The general multivariate MEMD has found applications in brain–computer interface [22,23], image processing [24,25], nuclear engineering [26] and system characterization [8].

In spite of the undoubted usefulness of the EMD algorithm and its variants, a rigorous theoretical description for the underlying algorithms is still lacking. To this end, the synchrosqueezing transform (SST or WSST) was proposed in [27]. It is a post-processing technique, originally based on the continuous wavelet transform (CWT), for the generation of highly localized TF representations of nonlinear and non-stationary signals. It reassigns the wavelet coefficients in scale or frequency by combining the coefficients that contain the same instantaneous frequencies, such that the resulting energy is concentrated around the instantaneous frequency curves of the modulated oscillations. The CWT, however, has a limited TF resolution, and because the CWT and STFT are both extensively used to analyse and process multicomponent signals, a natural extension of the univariate CWT-based SST (WSST) to the STFT setting was proposed in [28] and is referred to as STFT-based SST (FSST) (see appendix A.2 for more details). To identify oscillations common to multiple data channels, an extension of the univariate WSST to multivariate cases was proposed in [29], the so-called multivariate synchrosqueezing transform (MSST or W-MSST). It employs the WSST channel-wise to obtain the concentrated coefficients, and then estimates the multivariate instantaneous frequency by combining, for each frequency band, the instantaneous frequencies across all the channels using the joint instantaneous frequency (see [27,29–31] for more details). However, the performance of the W-MSST degrades with noise power, because: (i) the joint instantaneous frequency estimator is sensitive to noise and (ii) the CWT produces mathematical artefacts (additional noise)—wavelet coefficients which correspond to undesired frequency components. The additional noise (artefacts) generated by the W-MSST can be reduced by STFT-based MSST (F-MSST) (see appendix A.3 for more details).

To mitigate the aforementioned problems posed by both the intrinsic data association measures—poor time and frequency localization in IPS and loss of time information in ICoh—we, therefore, propose a highly localized data association measure which is achieved based on the combination of NA-MEMD and STFT-based SST and MSST (F-M/-SST) algorithms. The NA-MEMD is first employed to obtain physically meaningful intrinsic oscillations of a given multivariate signal. Owing to the resolution and noise problems in the CWT-based SST and MSST, we employ the STFT-based SST and MSST algorithms to generate highly localized TF representations of signal dependence between the intrinsic oscillations produced by the NA-MEMD. This procedure is referred to as the *intrinsic synchrosqueezing coherence* (ISC).

However, for certain scenarios, in the presence of

— collaborative tasks which take place over a long period of time,
— multiple occurrences of long and complex events during the tasks,
— physically meaningful and straightforward-to-interpret trends in the level of cooperation during the events being of interest, and
— prior knowledge of periods (i.e. frequency ranges) of the trends for physically meaningful and straightforward interpretation being unavailable,

it is imperative to have a data-driven data-association measure which empirically quantifies *intrinsic trends*, whereby only those components which contain physically meaningful interpretation can be combined. To this end, we propose an extension to the standard IPS, referred to as the *nested intrinsic phase synchrony* (N-IPS), which further decomposes time series of the degrees of synchrony between data channels obtained using the standard IPS into multiple scales of synchrony; then only certain scales which admit meaningful physical interpretation are empirically combined (i.e. summed), *without any prior knowledge of the frequencies of such scales*.

The aim of this study is to build upon the enhanced discrimination capability of the ISC and N-IPS data association metrics, in order to precisely identify physiological synchrony in frequency and time, using the ISC, and to empirically quantify physically meaningful intrinsic trends in the level of cooperation associated with events during the course of long cooperative tasks, using the N-IPS. We employ the ISC to precisely reveal the synchronized respiratory and HRV frequencies over time among a subset of bass singers of the Eric Whitacre Choir during 4 min and 40 s rehearsal and performance of the same musical score. The N-IPS is employed to empirically obtain physically meaningful trends in the levels of cooperation of: (i) subsets of soprano and bass singers of the Imperial College Chamber Choir during a 1 h evensong performance, through trends of synchrony in their respiratory and HRV signals and (ii) three pairs of catheterization laboratory team members (cardiology consultant, cardiology registrar, and physiologist) during a 2 h invasive coronary procedure, through trends of synchrony in their HRV signals.

## 2. Related work

In addition to our recently proposed data association measures, IPS and ICoh, there also exist several other synchrony measures. Cross-correlation is a simple measure of linear synchronization between two signals, and hence it cannot effectively deal with the nonlinear coupling behaviour, thus resulting in an undesired low value of correlation coefficient. Phase synchronization index (PSI) proposed in [32] is obtained by considering time-averaged phase difference between two signals, instead of considering the distribution of phase differences as employed in IPS and the proposed N-IPS (see §3.2 for more details). This technique can underestimate synchrony if the distribution of phase differences between two signals has more than one peak, and by averaging over time, phase differences can be cancelled out, resulting in an undesired low value of PSI. Note that the estimation of PSI in IPS, N-IPS and [32] is achieved via the calculation of instantaneous phase of the analytic signal generated using the Hilbert transform. Wavelet-based PSI was introduced in [33], whereby instantaneous phase is calculated by convolving each signal with a complex wavelet function and PSI is obtained in the same manner as in IPS and [32]. As a central frequency and a width of the wavelet function must be specified, this approach for estimating PSI is sensitive to phase synchrony only in a certain frequency band.

Synchrony can also be measured by means of information-theoric concepts [34], whereby the mutual information between two signals is defined as the indication of the amount of *information* of a signal which can be obtained by knowing the other signal and vice versa. The physical meaning or interpretation of synchrony quantified using this approach, however, does not exist.

General synchronization—the existence of a functional relationship between the systems generating the signals of interest—can be characterized by the conditional stability of the driven chaotic oscillator if the equations of the systems are known [35]. For real-world data, however, the model equations are typically unavailable. The non-parametric method of mutual false nearest neighbours [36], which is based on the technique of delay embedding and on conditional neighbours, therefore, has been proposed to characterize general synchronization, yet this technique might produce errors if the signals of interest have more than one predominant time scale [37]. Phase and general synchronization can also be quantified using the concept of recurrence quantification analysis, whereby two signals are deemed: (i) phase synchronized if the distances between the diagonal lines in their respective recurrence plots coincide and (ii) generally synchronized if their recurrence plots are very similar or approximately the same.

All of the described measures are limited to quantifying synchrony between the signals as a whole, and cannot yield TF representations of synchrony. Although such representations can be generated from the IPS algorithm, through the Hilbert transform, we have empirically found that for effective estimation of time-varying synchrony using IPS relatively long sliding windows should be used; hence its time localization is poor. Furthermore, a number of realizations of IPS must be performed for statistical relevance, thus inevitably blurring out TF representations of synchrony. On the other hand, the ISC proposed here generates highly localized TF representations of synchrony and is suitable for the analysis of synchrony in nonlinear and non-stationary multivariate signals.

## 3. Algorithm and background

The ISC and N-IPS algorithms proposed in this study are described below.

### 3.1. Intrinsic synchrosqueezing coherence

The proposed ISC is a data-association measure which exhibits precise time and frequency localization in the analysis of nonlinear and non-stationary multivariate signals. This is achieved through the combination of NA-MEMD, FSST and F-MSST algorithms. The NA-MEMD is first employed to decompose a given multivariate signal into a set of physically meaningful narrow-band intrinsic oscillations (IMFs). The FSST is next employed channel-wise to generate multiple univariate multicomponent TF planes with high localization in both time and frequency. The F-MSST is then used to construct multivariate highly localized TF representations. These univariate and multivariate TF representations are subsequently employed to generate TF representations of signal dependence (synchrony) in IMFs; see algorithm 1 for detail of the proposed ISC. The synchrosqueezing coherence index (SCI) ranges between 0 and 1, with 1 indicating perfect synchrony and 0 a non-synchronous state.

**Algorithm 1:** Intrinsic synchrosqueezing coherence.

**Input:** A multivariate signal **x**(*t*), **x**(*t*)=[*x*_1_(*t*),*x*_2_(*t*),…,*x*_*N*_(*t*)]^*T*^.

(i) Obtain IMFs via NA-MEMD, *c*_*n*,*k*_, where *n*=1,2,…,*N*, *k*=1,2,…,*K*, and *K* is the number of IMFs.
(ii) For each channel *n*, compute the Fourier spectra of IMFs and combine (sum) the IMFs governing the frequency band of interest, *IMFs*_*n*_.
(iii) Apply FSST channel-wise to the combined IMFs, *IMFs*_*n*_, to generate *N* univariate TF representations, *T*_*n*_( *f*,*t*), where *n*=1,2,…,*N*.
(iv) Apply F-MSST to the combined IMFs between channels *i* and *j*, *IMFs*_*i*_ and *IMFs*_*j*_, where *i*=1,2,…,*N*, *j*=1,2,…,*N*, and *i*≠*j*, to generate a multivariate TF representation of the two channels, *T*_*i*,*j*_( *f*,*t*).
(v) TF representation of the degree of signal dependence (synchrony) in frequency and time between channels *i* and *j* is then determined via the SCI, given by
  3.1 $${SCI}_{i,j}(\;f,t)=\frac{\sqrt{\frac{|T_{i}(\;f,\;t)|\cdot |T_{j}(\;f,\;t)|}{|T_{i,j}(\;f,\;t)|}}}{\underset{f,t}{\text{max}}\left(\sqrt{\frac{|T_{i}(\;f,\;t)|\cdot |T_{j}(\;f,\;t)|}{|T_{i,j}(\;f,\;t)|}}\right)};\forall f,\forall t$$
(vi) Perform 30 realizations of NA-MEMD, repeat steps (i)–(v) for each realization, and average the 30 TF representations of signal dependence between channels *i* and *j*, *SCI*_*i*,*j*_( *f*,*t*), in order to obtain a highly localized TF representation of synchrony.

### 3.2. Nested intrinsic phase synchrony

The IPS was originally proposed in the so-called *intrinsic multi-scale analysis* framework in [8] and generalizes standard phase synchrony by equipping it with the ability to operate at the intrinsic scale level. It employs NA-MEMD to decompose a given multivariate signal into its narrow-band intrinsic oscillations (IMFs), which makes it possible to quantify the temporal locking of the phase information in IMFs using the standard PSI.

We here introduce an extension to the IPS, referred to as N-IPS, for the empirical quantification of physically meaningful and straightforward-to-interpret trends in phase synchrony. The N-IPS first employs the conventional IPS to quantify the intrinsic phase relationship in IMFs, and further decomposes the resulting time series of IPS into multiple scales, whereby only certain scales which contain physically meaningful and straightforward-to-interpret information are then empirically combined (summed), as outlined in algorithm 2. Note that trends in synchrony obtained using the N-IPS algorithm can be negative, because IMFs of synchrony which contain a positive offset in the raw PSI values can be neglected. In such circumstances, the baseline for the trends in synchrony obtained using N-IPS is an imperative, because it is used to judge whether the trends in synchrony are significant or not—they are deemed significant if above the baseline.

**Algorithm 2:** Nested intrinsic phase synchrony.

**Input:** A multivariate signal **x**(*t*), **x**(*t*)=[*x*_1_(*t*),*x*_2_(*t*),…,*x*_*N*_(*t*)]^*T*^.

(i) Obtain IMFs via NA-MEMD, *c*_*n*,*k*_, where *n*=1,2,…,*N*, *k*=1,2,…,*K*, and *K* is the number of IMFs.
(ii) For each channel *n*, calculate the instantaneous phases for the IMFs through the analytic signals generated using the Hilbert transform.
(iii) Calculate phase difference between the instantaneous phases for the IMFs of channels *i* and *j*, *ϕ*_*i*,*j*_(*t*), where *i*=1,2,…,*N*, *j*=1,2,…,*N*, and *i*≠*j* .
(iv) Phase synchrony between channels *i* and *j* is then defined in terms of the deviation from perfect synchrony via the PSI [38], given by
  3.2 $$\rho_{i,j}(t)=\frac{S_{max}-S}{S_{max}},$$
  where $S=-\sum_{m=1}^{M}p_{m}\ln p_{m}$ is the Shannon entropy of the distribution of phase differences $\phi_{i,j}(t-\frac{W}{2}:t+\frac{W}{2})$ within a window of length *W*, *M* is the number of bins within the distribution of phase differences, and *p*_m_ is the probability of $\phi_{i,j}(t-\frac{W}{2}:t+\frac{W}{2})$ within the *m*th bin. The maximum entropy *S*_*max*_ is given by
  3.3 $$S_{max}=0.626+0.4\ln(W-1).$$
(v) Obtain IMFs of the phase synchrony between channels *i* and *j*, *ρ*_*i*,*j*_(*t*), via NA-MEMD, *c*_*ρ*_*i*,*j*_,*l*_, *l*=1,2,…,*L*, and *L* is the number of IMFs of the phase synchrony.
(vi) Empirically combine (sum) certain IMFs of the phase synchrony, *c*_*ρ*_*i*,*j*_,*l*_, which contain physically meaningful and straightforward interpretation.

## 4. Applications

We shall now demonstrate applications of the proposed ISC algorithm in: (i) estimating degrees of synchrony in a linear bivariate signal, (ii) estimating degrees of synchrony in a nonlinear narrow-band bivariate signal and (iii) the precise identification of physiological synchrony among choir members. We also demonstrate applications of the N-IPS algorithm in large-scale analyses for the empirical quantification of the levels of cooperation in: (i) choir singing and (ii) performing a surgical procedure.

### 4.1. Estimating degrees of synchrony in a linear bivariate signal

The utility of the proposed ISC algorithm, a combination of NA-MEMD and STFT-based M/-SST (F-M/-SST) algorithms, is demonstrated over the task of estimating degrees of synchrony in a linear bivariate signal against the standard IPS algorithm, which is a combination of NA-MEMD and phase synchrony, and other five combinations of algorithms for performing steps (i)–(iv) in algorithm 1. These combinations are:

— FIR band-pass filter (BPF) and CWT-based M/-SST (W-M/-SST);
— FIR BPF and STFT-based M/-SST (F-M/-SST);
— IIR BPF and CWT-based M/-SST (W-M/-SST);
— IIR BPF and STFT-based M/-SST (F-M/-SST);
— NA-MEMD and CWT-based M/-SST (W-M/-SST).

For validating purposes, to resemble real-world physiological signals, the linear bivariate signal consisted of sinusoidal oscillations corrupted by additive 1/*f* noise and a low-frequency trend of magnitude *A* oscillating at 0.01 Hz, *A*_0.01_, given by

**Figure 1.**
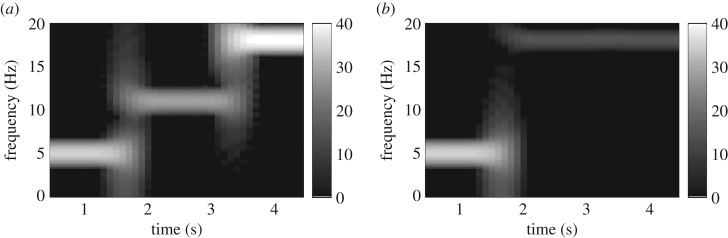
Standard TF spectrograms of the clean linear bivariate signal. (*a*) First channel consisted of three sinusoids of frequencies 5 Hz, 11 Hz and 18 Hz. (*b*) Second channel consisted of two sinusoids of frequencies 5 Hz and 18 Hz. There were, therefore, both strong and weak signal dependence at 5 Hz and 18 Hz at different time instants.

4.1 $$\begin{array}{rl} x_{1}(t) & =A_{0.01}+k*\cos(2\pi f(t)t)+n_{1}(t),\\ f & (t)=\left\{ \begin{array}{l} f_{1};t=0\;s,\ldots ,1.667\;s\\ f_{2};t=1.667\;s,\ldots ,3.334\;s\\ f_{3};t=3.334\;s,\ldots ,5\;s \end{array}\right.\\ k & =\left\{ \begin{array}{l} k_{1};t=0\;s,\ldots ,1.667\;s\\ k_{2};t=1.667\;s,\ldots ,3.334\;s\\ k_{3};t=3.334\;s,\ldots ,5\;s \end{array}\right.\\ x_{2}(t) & =A_{0.01}+k*\cos(2\pi f(t)t)+n_{2}(t),\\ f(t) & =\left\{ \begin{array}{l} f_{1};t=0\;s,\ldots ,1.667\;s\\ f_{3};t=1.667\;s,\ldots ,5\;s \end{array}\right.\\ \text{and}\;k & =\left\{ \begin{array}{l} k_{1};t=0\;s,\ldots ,1.667\;s\\ k_{4};t=1.667\;s,\ldots ,5\;s \end{array}\right. \end{array}\}$$

where *A*_0.01_=5, *f*_1_=5 Hz, *f*_2_=11 Hz, *f*_3_=18 Hz, *k*_1_=1, *k*_2_=0.5, *k*_3_=2, *k*_4_=0.1 and the sampling frequency *f*_s_=200 Hz. The SNR of the first channel corrupted by additive 1/*f* noise *n*_1_ was 10 dB, while the SNR of the second channel governed by the ‘physiological’ 1/*f* noise *n*_2_ was varied between −10 dB and 20 dB. Figure 1 shows the spectrograms of the clean linear bivariate signal without the low-frequency trend estimated using the standard STFT. In this bivariate signal, there was strong synchrony between the two channels at 5 Hz from 0 s to 1.667 s, and weak synchrony at 18 Hz from 3.334 s to 5 s. The bivariate signal was decomposed using NA-MEMD with 10 adjacent WGN channels. Three IMFs of each of the two channels which governed the frequency band of 4–19 Hz were combined and averaged over 30 realizations of NA-MEMD. Alternatively, the bivariate signal was band-pass filtered using an FIR BPF of order 50 with the passband set to 4–19 Hz, a bandwidth similar to the combined IMFs. A Butterworth IIR BPF of order 3 with the same passband was also applied to the bivariate signal. The different filtered bivariate signals obtained using the NA-MEMD, FIR BPF and IIR BPF were then fed into the W-M/-SST and F-M/-SST algorithms to generate TF representations of signal dependence (synchrony) calculated using equation (3.1) in algorithm 1. Phase synchrony estimation was also employed to estimate phase relationship in the filtered bivariate signal obtained using NA-MEMD—IPS.

**Figure 2.**
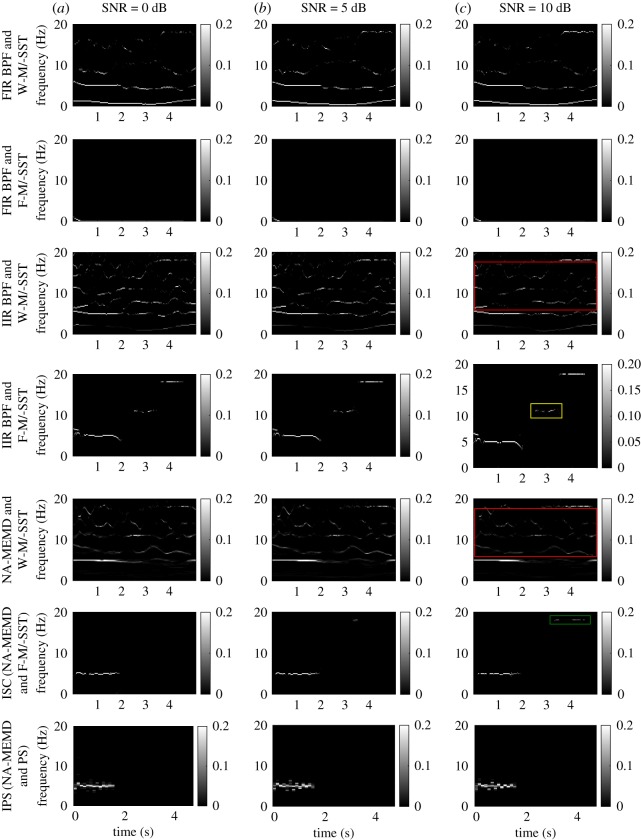
TF representations of the degrees of synchrony in a synthetic linear bivariate signal in figure 1 estimated using different combinations of the considered algorithms. The SNR of the second channel governed by 1/*f* noise was set to 0 dB, 5 dB and 10 dB (*a*, *b* and *c*, respectively). Observed mathematical artefacts produced by CWT and highlighted in the red boxes. Undesired synchrony at 11 Hz was produced by the IIR BPF and F-M/-SST as shown in the yellow box. The ISC algorithm, NA-MEMD and F-M/-SST, performed the best due to no spurious synchrony and the identified weak synchrony at 18 Hz (see the synchrony in the green box).

Figure 2 shows TF representations of degrees of synchrony in the linear bivariate signal estimated using the different combinations of algorithms, where the SNR of the second channel was set to 0 dB, 5 dB and 10 dB (results from the other SNRs are not shown). The W-M/-SST combined with either FIR BPF, IIR BPF or NA-MEMD, produced noticeable spurious synchrony (see the first, third and fifth rows), because CWT introduced mathematical artefacts (noise), and consequently they were localized by the joint frequency estimator in W-MSST. As the 0.01 Hz trend added to the signal was not filtered out completely using the FIR BPF and the remaining trend was localized by F-M/-MSST, the SCI values at this frequency consequently dominated the TF representations of degrees of synchrony (second row). Undesired synchrony at *f*_2_ was produced by the combination of IIR BPF and F-M/-SST (see fourth row). Using IPS (last row), the degrees of synchrony estimated at *f*_1_ were not highly localized, spreading around *f*_1_, and the estimation of weak synchrony at *f*_3_ was not achieved. As desired, the proposed ISC algorithm (NA-MEMD and F-M/-SST, sixth row) did not produce spurious synchrony, yielded desired synchrony at *f*_1_ and achieved the estimation of weak synchrony at *f*_3_ at a high SNR.

### 4.2. Estimating degrees of synchrony in a nonlinear narrow-band bivariate signal

We next evaluated the performance of the seven combinations of algorithms in the task of estimating synchrony in a nonlinear narrow-band bivariate signal extracted from a nonlinear wide-band bivariate AM/FM signal corrupted by additive WGN, given by

4.2 $$\begin{array}{rl} x_{1}(t) & =((1+M_{\mathrm{AM}}*\cos(2\pi f_{m}(t)))*A_{1})\\ & \;*\sin(2\pi f_{c}(t)+(A_{1}*M_{\mathrm{FM}}*\sin(2\pi f_{m}(t))))+n(t)\\ \text{and}\;x_{2}(t) & =((1+M_{\mathrm{AM}}*\cos(2\pi f_{m}(t)))*A_{2})\\ & \;*\sin(2\pi f_{c}(t)+(A_{2}*M_{\mathrm{FM}}*\sin(2\pi f_{m}(t))))+n(t), \end{array}\}$$

where *M*_AM_ denotes amplitude modulation index, *M*_FM_ frequency modulation index, *f*_*c*_ carrier frequency, *f*_m_ baseband frequency, *A*_1_ and *A*_2_ scalars which define signal magnitude, and *n*(*t*) the added WGN. In this example, *M*_AM_=0.5, *M*_FM_=20, *f*_*c*_=500 Hz, *f*_m_=11 Hz, *A*_1_=1, *A*_2_=0.5 and the sampling frequency *f*_s_=1 kHz. The SNRs of both the channels were varied between −10 dB and 20 dB. The NA-MEMD was employed to extract a narrow-band bivariate signal of 7–15 Hz (*f*_m_±4 Hz) from the corrupted wide-band bivariate signal. The corrupted signal was also alternatively band-pass filtered to 7–15 Hz by FIR and IIR BPFs with the same orders as in the previous example. The degrees of synchrony in the filtered narrow-band bivariate signals were then estimated as previously described.

**Figure 3.**
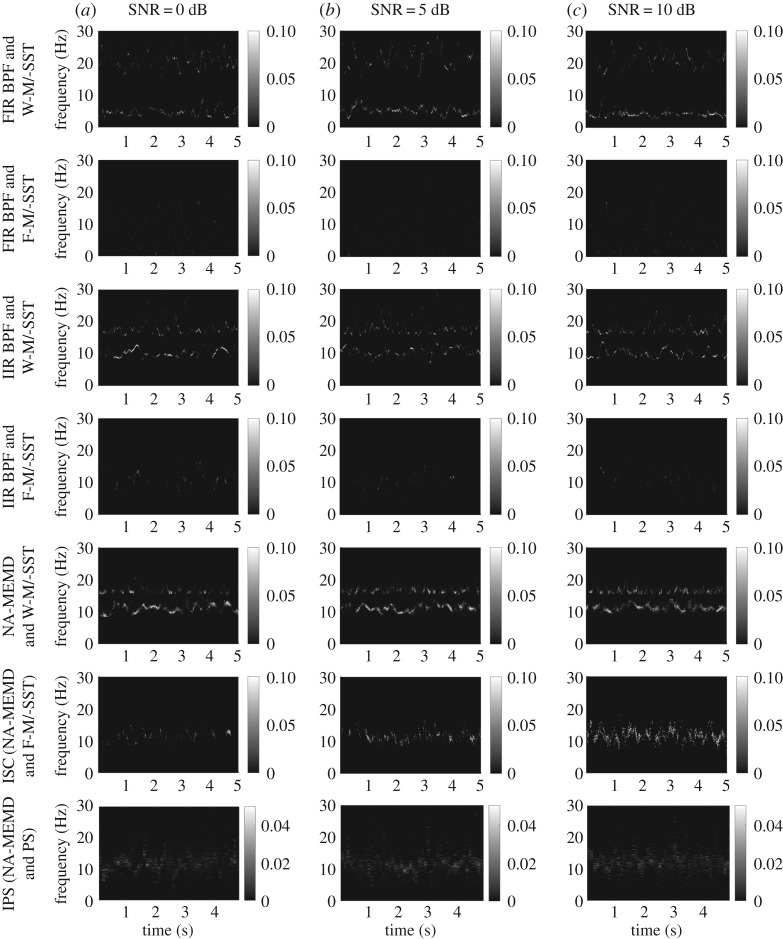
TF representations of the degrees of synchrony in a synthetic nonlinear bivariate signal estimated using different combinations of algorithms. The SNR of the second channel governed by WGN noise was set to 0 dB, 5 dB and 10 dB (*a*, *b* and *c* panels, respectively). The ISC algorithm, NA-MEMD and F-M/-SST, performed the best due to the identified synchrony in the band of interest and no spurious synchrony outside of this band.

Figure 3 shows TF representations of degrees of synchrony in the nonlinear narrow-band bivariate signal estimated using the different combinations of algorithms, where the SNRs of both the channels were set to 0 dB, 5 dB and 10 dB (results from the other SNRs are not shown). Using W-M/-SST combined with either FIR BPF, IIR BPF or NA-MEMD, there exist noticeable spurious synchronies outside the band of interest (see the first, third and fifth rows). Synchrony in the band of interest was not exhibited using the combination of FIR BPF and F-M/-SST (second row), and was poorly estimated using the combination of IIR BPF and F-M/-SST (see fourth row). The proposed ISC algorithm (NA-MEMD and F-M/-SST, sixth row) did not produce spurious synchrony outside the band of interest, and exhibited the desired synchrony at high SNRs, outperforming IPS (last row) at 5 dB and 10 dB SNRs.

**Figure 4.**
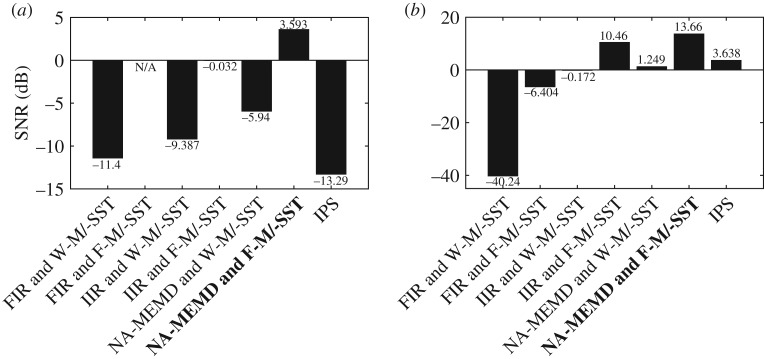
Average SNRs of TF representations of signal dependence estimated using different algorithms. (*a*) Average SNRs of TF representations of synchrony in the synthetic linear bivariate signal. (*b*) Average SNRs of TF representations of synchrony in the synthetic nonlinear bivariate signal.

Figure 4 shows average SNRs of the TF representations of synchrony produced by the seven combinations. The degrees of synchrony estimated at the frequencies *f*_1_ and *f*_3_ in the linear bivariate signal and those in the band of interest in the nonlinear bivariate signal were deemed ‘signals’, and the degrees of synchrony outside these were deemed ‘noise’. Note that the SNR of the FIR and F-M/-SST in the first task could not be computed, because no ‘signal’ was present in its TF representations. The Z-test at a significance level of 0.01 was performed, in order to reveal statistical differences in the SNRs between the proposed ISC algorithm and the second best combination in both the tasks. Average SNRs of the proposed ISC algorithm (3.593 dB and 13.66 dB) were 3.625 dB and 3.2 dB significantly higher (*p*-value=0.0001 and 0.0004, respectively) than those (−0.032 dB and 10.46 dB) of the second best combination, which is the IIR BPF and F-M/-SST, in estimating degrees of synchrony in, respectively, the synthetic linear and nonlinear bivariate signals. The ISC algorithm outperformed the others in both of the tasks, because essentially it did not produce spurious synchrony, making it the most reliable among them. It should also be noted that: (i) both FIR and IIR filters generally introduce several problems such as group delay and nonlinear phase response; (ii) these filters are not data-driven and not designed specifically for nonlinear and non-stationary signals as opposed to NA-MEMD; and (iii) CWT produces mathematical artefacts which degrade the performance of W-MSST.

### 4.3. Highly localized heart rate variability- and respiration-based choral synchrony analysis

#### 4.3.1. Motivation

Our recent work [1,39] has shown that during choral singing, HRV and respiration of a subset of five singers synchronized, with an increase from the rehearsal to the performance *on the average*, and the physiological synchrony in both situations, *as a function of frequency*, dominated the LF band. This exemplifies the better coordination among the singers under the situation that was more stressful. That study, however, examined the average level of cooperation by averaging the PSI and coherence of the respiratory and HRV signals of three different voices (one tenor, one soprano and three bass singers) over time. This did not allow for the quantification of the level of cooperation of specific voices and at different time instants where the tempo of the musical score varied. In addition, a highly localized TF representation of synchrony in the respiration and HRV signals for thorough investigation of the frequential and temporal dynamics of the physiological synchrony was not available.

To this end, we here employ: (i) IPS to reveal the degrees of time-varying synchrony in HRV and respiration among only the three bass singers, since as the same voice they performed long or short inhalation or exhalation almost exactly at the same time over the course of the 4 min and 40 s rehearsal and performance; and (ii) ISC to precisely quantify synchrony in the physiological signals in both time and frequency. Furthermore, the time-varying synchrony estimated using IPS was used to verify patterns of temporal dynamics and time localization of the TF representations of the physiological synchrony obtained using the proposed ISC algorithm.

#### 4.3.2. Data acquisition and synchrony analysis

Respiratory and ECG signals were recorded from a subset of three bass singers of the 18-member Eric Whitacre’s Choir during 4 min and 40 s periods of a low-stress rehearsal and a high-stress public performance at Union Chapel, London, UK. Respiration of each participant was recorded using a custom-made respiration belt placed around the chest. For all participants, the ECG was recorded with three electrodes placed on the skin, just below the collar bone. The respiration belt and the electrodes were connected to an eight-channel portable biosignal data logger powered by a rechargeable coin cell battery. The data logger sampled the signals at 1 kHz and saved the respiratory and ECG data onto a micro-SD card. The data logger also recorded timestamps onto the micro-SD card in order to guarantee the synchronization between the devices. The respiratory signals were downsampled to 4 Hz, and the HRV was estimated from the ECG data by band-pass filtering between 8 Hz and 30 Hz, and the subsequent R-peak detection to obtain the RR-interval (i.e. HRV) time series with a sampling frequency of 4 Hz [40].

The respiratory (or HRV) signals of the three bass singers during both the rehearsal and the performance were used to form three-channel data which was decomposed using NA-MEMD with 10 adjacent WGN channels. The IMFs with indices 3–6 produced by the NA-MEMD of the three-channel multivariate HRV (or respiratory) signal of the bass singers contained the physically meaningful frequency range 0.04 Hz to 0.4 Hz, that is, exactly the LF/HF frequency band of HRV. The full band of interest in both the HRV and respiration data was produced by summing up the corresponding IMFs, in order to obtain the desired scale in data. The time-varying PSI values among the singers were obtained by averaging PSI values calculated from three combined-IMF pairs of the three data channels (pair 1: first and second bass singers, pair 2: first and third bass singers, pair 3: second and third bass singers), whereby the PSI values for each pair were computed using 20 s sliding windows with 18 s overlap (2 s increment) for the respiratory signals, and 40 s sliding windows with 36 s overlap (4 s increment) for the HRV signals. The PSI indices between the combined IMFs of the noise channels were also estimated in order to provide the PSI of random signals as a baseline.

The ISC was performed on each of the same three combined-IMF pairs of the three data channels of the respiratory (or HRV) signals to obtain three TF representations of synchrony in each of the signals for all the pairs. The three TF representations were next averaged in order to obtain a TF representation of synchrony in the respiratory (or HRV) signals among the bass singers.

#### 4.3.3. Results of the analysis

**Figure 5.**
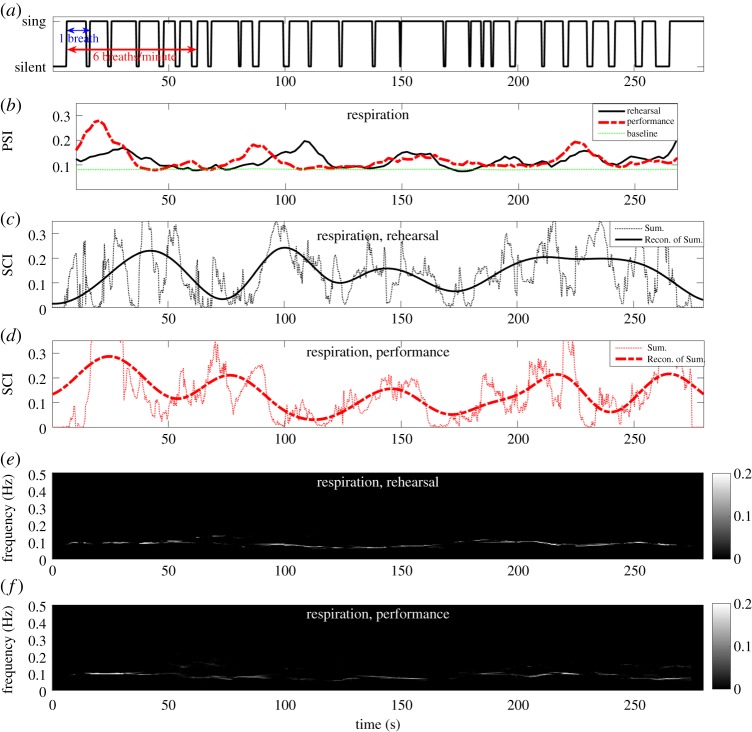
Intrinsic synchrony in respiratory signals among the bass singers during the rehearsal and the performance estimated using IPS and STFT-based ISC. (*a*) Singing timing of the bass singers. (*b*) The PSI of the respiratory signals. (*c*) The sum of SCI values across frequencies of the respiratory signals during the rehearsal, and its reconstruction from IMFs with indices 8–11. (*d*) The sum of SCI values across frequencies of the respiratory signals during the performance, and its reconstruction from IMFs with indices 8–11. (*e*) The SCI of the respiratory signals during the rehearsal. (*f*) The SCI of the respiratory signals during the performance. For brevity, the sum of SCI values are denoted by Sum., and the reconstruction of the sum of SCI values from IMFs with indices 8–11 by Recon. of Sum. Observe: (i) similar patterns of temporal dynamics between the Recon. of Sum. and the corresponding PSIs; and (ii) time instants of peaks in the Recon. of Sum. are close to those of the peaks in the corresponding PSIs.

**Figure 6.**
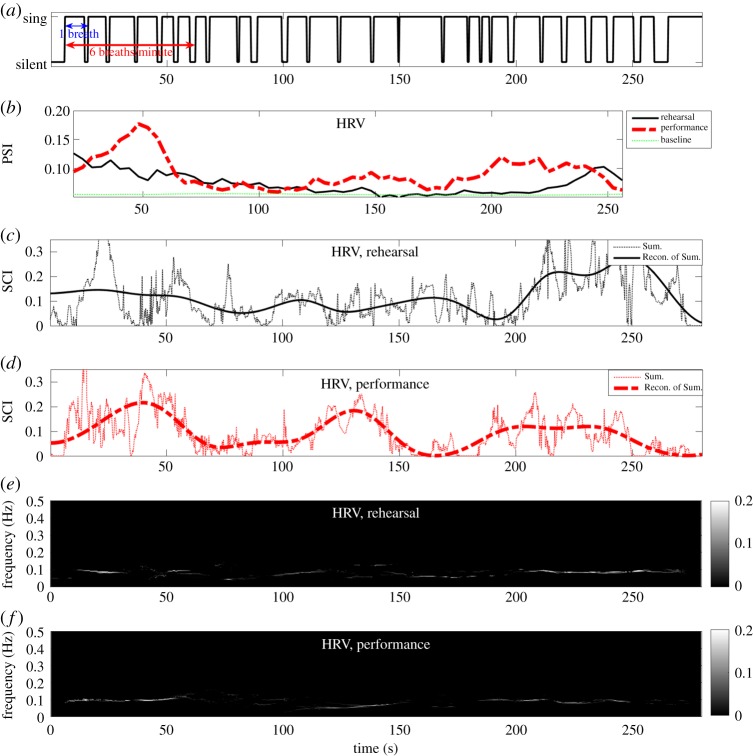
Intrinsic synchrony in HRV signals among the bass singers during the rehearsal and the performance estimated using IPS and STFT-based ISC. (*a*) Singing timing of the bass singers. (*b*) The PSI of the HRV signals. (*c*) The sum of SCI values across frequencies of the HRV signals during the rehearsal, and its reconstruction from IMFs with indices 8–11. (*d*) The sum of SCI values across frequencies of the HRV signals during the performance, and its reconstruction from IMFs with indices 8–11. (*e*) The SCI of the HRV signals during the rehearsal. (*f*) The SCI of the HRV signals during the performance. For brevity, the sum of SCI values are denoted by Sum., and the reconstruction of the sum of SCI values from IMFs with indices 8–11 by Recon. of Sum. Observe: (i) similar patterns of temporal dynamics between the Recon. of Sum. and the corresponding PSIs; and (ii) time instants of the peaks in the Recon. of Sum. are close to those of the peaks in the corresponding PSIs.

Figures 5*a* and 6*a* show the timings of long or short exhalation of the bass singers when they performed in unison, and when they remained silent or inhaled. Figures 5*b* and 6*b* show degrees of synchrony in, respectively, the respiratory and HRV signals during the rehearsal and the performance, estimated using IPS with 30 realizations of NA-MEMD. Observe that the respiratory synchrony in both situations (figure 5*b*) reached their peaks approximately during a series of long exhalation. Figures 5*e*–*f* and 6*e*–*f* show TF representations of, respectively, respiratory and HRV synchrony during the rehearsal and the performance estimated using the proposed STFT-based ISC. In both situations, the respiratory synchrony (figure 5*e*–*f*) was highly localized at 0.1 Hz due to a series of long exhalations they had to perform in unison. This exemplifies that the singers were constrained by the musical score to breathe in unison at a very slow rate of six breaths per minute (breaths per minute=60×breathing frequency). This rate is remarkably slower than the normal breathing rate in adults, which varies between 12 and 18 breaths per minute [41]. The HRV synchrony (figure 6*e*–*f*) was dominant in the LF band of HRV, 0.04–0.15 Hz. This conforms with our recent finding in [1], where ICoh revealed a large proportion of coherence in the HRV signals in the LF band.

The sums of SCI values across frequencies as a function of time of the respiratory and HRV signals during both the rehearsal and the performance are, respectively, shown in figures 5*c*–*d* and 6*c*–*d* (see thin dotted lines). Observe that the sums of SCI values of both the signals varied dramatically during both situations, indicating that the proposed ISC algorithm effectively yielded highly localized time-varying respiratory and HRV synchrony. To perform straightforward verification of patterns of temporal dynamics and time localization of the relatively ‘faster’ temporal dynamics of the sums of SCI values against the ‘slow’ temporal dynamics of the corresponding PSIs shown in figures 5*b* and 6*b*, the sums of SCI values of both the signals during both situations were used to form four-channel data which was decomposed using MEMD. We empirically found that the multivariate IMFs with indices 1–7 of the four-channel data could be deemed ‘fast oscillatory components’, while the multivariate IMFs with indices 8–11 ‘slow oscillatory components’, and that the combinations of the multivariate IMFs in the latter set yielded the reconstructions of the sums of SCI values which exhibited: (i) ‘slow’ temporal dynamics similar to the ‘slow’ temporal dynamics of the corresponding PSIs for both the signals and in both situations; and (ii) time instants of peaks in the reconstructions of the sums of SCI values of both the signals and in both situations being close to those of the peaks in the corresponding PSI in figures 5*b* and 6*b*. Observe similar patterns of temporal dynamics and close positions of the peaks between: (i) the solid lines in figure 5*b*,*c*; (ii) the thick broken lines in figure 5*b*,*d*; (iii) the solid lines in figure 6*b*,*c* and (iv) the thick broken lines in figure 6*b*,*d*.

**Figure 7.**
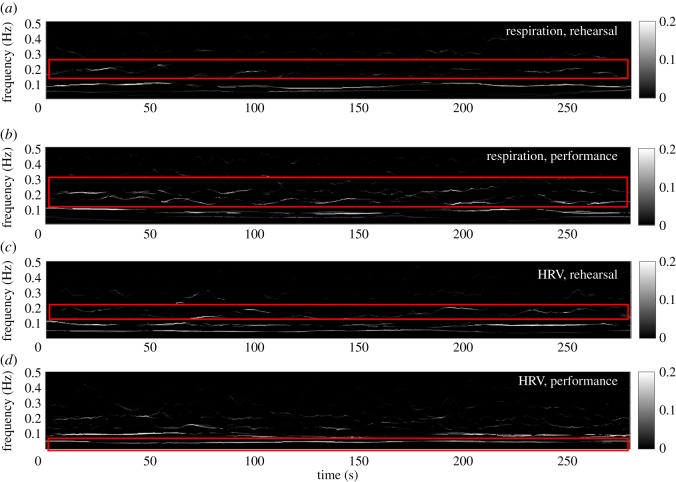
Intrinsic synchrony in respiratory and HRV signals among the bass singers during the rehearsal and performance estimated using CWT-based ISC. (*a*) The SCI of the respiratory signals during the rehearsal. (*b*) The SCI of the respiratory signals during the performance. (*c*) The SCI of the HRV signals during the rehearsal. (*d*) The SCI of the HRV signals during the performance. Observe mathematical artefacts shown in the red boxes. These are introduced by the CWT.

We also examined the degrees of synchrony in the bass singers’ respiratory and HRV signals during both the situations using CWT-based ISC, which combines the conventional CWT-based SST and MSST algorithms, as shown in figure 7*a*–*d*. As the joint frequency estimator in the CWT-based MSST is sensitive to noise [29] and CWT produced mathematical artefacts (additional noise), the TF representations produced by the CWT-based ISC exhibited notable spurious synchrony at several frequencies.

### 4.4 Large-scale heart rate variability- and respiration-based choral synchrony analysis

#### 4.4.1. Motivation

In addition to §4c where the degrees of synchronization in the respiratory and HRV signals were highly localized in frequency and time using the proposed ISC algorithm, it is equally important to identify the dynamics of the level of cooperation in response to events during the course of long cooperative tasks. Therefore, quantifying trends in synchrony, i.e. the level of cooperation, in choir singers during an evensong performance, which typically takes place over a long period of time and comprises several events (singing and pausing), is the subject of this study.

Owing to the nature of an evensong performance, where all the events have different lengths, time series of synchrony in singers’ respiratory and HRV signals typically exhibit nonlinear behaviour (i.e. deformed wave profiles). Such time series of synchrony can be empirically decomposed using a second NA-MEMD into a set of physically meaningful nonlinear components (IMFs) of the synchrony without prior knowledge of the frequency ranges (periods) of trends. In this way, we obtain physically meaningful and straightforward interpretation in response to the events, as opposed to low- or band-pass filtering, where cut-off frequencies are rigid, must be determined and specified beforehand, and are person-specific (unlike the proposed approach), which causes physically meaningful synchrony events to be missed. To this end, the proposed N-IPS algorithm was employed to empirically obtain physically meaningful and straightforward-to-interpret trends in the degrees of synchronization in: (i) respiratory signals among a subset of three soprano singers and a subset of three bass singers; and (ii) HRV signals among a subset of five soprano singers and a subset of four bass singers, of the 20-member Imperial College Chamber Choir during a 1 h evensong performance at St Martin-in-the-Fields church at Charing Cross, London, UK.

#### 4.4.2. Data acquisition and synchrony analysis

The data were acquired in the same manner as in §4.3, except for the group and number of singers and the duration of the recording; for this analysis the respiratory and HRV signals were recorded from 10 min before the performance, 1 h of the performance and 10 min after the performance.

The respiratory (or HRV) signals of the soprano and bass singers were used to form multichannel data which was decomposed using NA-MEMD with 10 adjacent WGN channels. The IMFs which were produced by the NA-MEMD with indices 3–6 of the multivariate HRV (or respiratory) signal of the singers and contained the physically meaningful frequency range 0.04 Hz to 0.4 Hz (the LF/HF frequency band of HRV) were then summed up to obtain the desired scale in data. The PSI estimation was next performed in the same manner as in §4.3. The time-varying PSI indices between the combined IMFs of the signal and noise channels (baseline) were next used to form multichannel data which were further decomposed using NA-MEMD with 10 adjacent WGN channels. Certain IMFs of the multivariate synchrony (synchrony-IMFs) produced by the NA-MEMD (IMFs 5–7 for the respiratory synchrony, 5–6 for the HRV synchrony) were then combined, because the combinations of the synchrony-IMFs admit capturing of physically meaningful and interpretable trends in the respiratory and HRV synchrony; that is, temporal dynamics of the combinations of the synchrony-IMFs closely resemble the timings of the songs and the pauses, where increases (during songs) and decreases (during pauses) in trends of the physiological synchrony, i.e. the level of cooperation, were physiologically expected to be observed. The other IMFs, on the other hand, were neglected due to no clear physical meaning or straightforward interpretation; their temporal dynamics did not resemble the timings of the songs and the pauses, and distinct increases and decreases in trends of the synchrony during the songs and the pauses, respectively, cannot be observed.

#### 4.4.3. Results of the analysis

**Figure 8.**
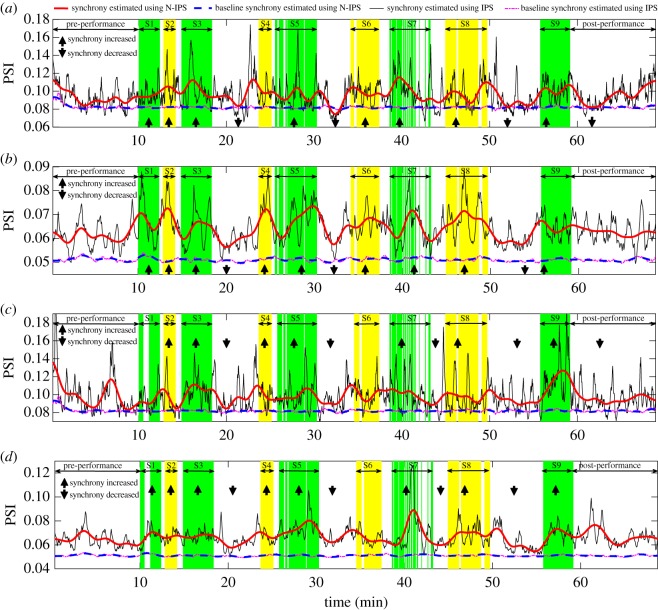
Time-varying synchrony in the sopranos’ and basses’ respiratory and HRV signals. (*a*) Respiratory PSI among the sopranos. (*b*) HRV PSI among the sopranos. (*c*) Respiratory PSI among the basses. (*d*) HRV PSI among the basses. Degrees of raw synchrony (thin solid lines) and the corresponding baselines (thin broken lines) were estimated using IPS. Trends of the raw synchrony (thick solid lines) and the corresponding baselines (thick broken lines) were estimated using N-IPS. Time instants when the singers sang during the nine songs (S1–S9) are depicted by the shaded areas. Observe increases in the synchrony of singers’ physiological responses during most of the songs depicted by the upward pointing arrows. These are due to their breathing rhythms and HRV being modulated by the demands of pieces of music.

Figure 8*a*–*d* shows physically meaningful trends in synchrony in the sopranos’ and basses’ respiratory and HRV signals, estimated from 30 realizations of NA-MEMD. Observe that all the ‘raw’ synchrony obtained using the standard IPS algorithm (solid thin lines) varied dramatically during the course of events (singing and pausing), thus being less amenable to physical interpretation. Trends in the synchrony obtained using the proposed N-IPS algorithm, on the other hand, exhibited smooth variations of synchrony during the events, and smooth transitions between them. This remarkably offers more physically meaningful and interpretable results; that is, the levels of cooperation markedly increased during most of the songs (depicted by the upward pointing arrows), and decreased during the long pauses (the downward pointing arrows). Variations in the degrees of synchrony (levels of cooperation) were because the breathing rhythms, and by virtue of RSA the cardiac activities too, of the singers were modulated by the pieces of music performed, thus exhibiting stronger dynamic coupling reflected in increases in the synchrony of their physiological responses. During the pauses, however, their breathing rhythms were not dictated by any piece of music, thus resulting in decreases in the synchrony. It should be noted that the HRV and respiratory signals were recorded from an amateur choir. Their breathing rhythms, therefore, may not have been perfectly synchronized. This is reflected by the transitional periods of the trends in synchrony between the songs and pauses occasionally not being perfectly aligned with the exact transitional timings between the songs and pauses. It also must be emphasized that we here focused on the empirical quantification of physically meaningful and straightforward-to-interpret trends in synchrony, which is a first step towards prediction.

### 4.5. Large-scale heart rate variability-based surgical synchrony analysis

#### 4.5.1. Motivation

Invasive cardiology procedures are performed on a daily basis in dedicated centres. Every patient with chest pain and diagnosed myocardial infraction goes for primary coronary angioplasty, which is an established management giving the best outcomes in terms of recovery and survival. Real-life HRV recording and simultaneous acquisition of its changes between team members involved in the procedure will give an objective tool for assessment of the team performance and efficiency. Empirical evidence supports the view that the quality of surgical performance depends not only the technical skills of the surgical teams, but also on good collaboration and effective teamwork [42]. Failures in non-technical skills in the operating room have been frequently implicated in more frequent errors, adverse events in surgical patients and longer operative times [43,44]. Non-technical skills also have a direct impact on the technical performance of surgical teams [45].

Multidisciplinary interventional cardiology catheterization laboratory (Cath Lab) teams are composed of an interventional cardiology consultant, a cardiology registrar, a nurse, a physiologist and a radiographer, each with a different background in terms of training and competencies. These team members are expected to work and function in full synchrony to achieve optimal outcomes. Close cooperation between physicians performing the procedure (cardiology consultant and cardiology registrar) and physiologist outside the suite who record and monitor pressures during coronary intervention is essential.

The case presented in the manuscript is part of a pilot study, which aims to show that a team, whose members work together on a daily basis and have developed mutual understanding and trust, can respond quicker and more efficiently to unexpected situations. Common experience of team members has been found to play a critical role in good collaboration in the operating theatre and is increasingly recognized as an important mechanism for enhancing the safety of delivered healthcare services. It has been shown that team familiarity has a threefold greater impact on the duration of the surgical procedure than the experience of the main surgeon [46].

#### 4.5.2. Data acquisition and synchrony analysis

ECG signals were recorded from a Cath Lab team starting 10 min before, during and after a percutaneous coronary intervention procedure, which was 1 h and 56 min long, and was carried out in the Cath Lab at Hammersmith Hospital, Imperial College Healthcare NHS Trust, London. During the procedure, team members were closely observed by an independent observer, who noted times and types of any glitches that occurred. Based on the published literature, we have divided possible glitches into 13 categories [47]. The electrode placement for ECG recording, data acquisition, HRV estimation and PSI estimation were carried out in the same manner as in §4.3. Similarly to §4.4, a second NA-MEMD was employed to obtain trends in synchrony in the surgeons’ HRV signals in order to cater for: (i) the nonlinear behaviour of the synchrony measure and (ii) the lack of prior knowledge of the frequency ranges (periods) of trends, thus providing physically meaningful and straightforward interpretation in response to glitches. The time-varying PSIs between IMFs of the HRVs and noise channels (baseline) were used to form multichannel data, which were further decomposed using NA-MEMD with 10 adjacent WGN channels. The IMFs produced by the NA-MEMD with indices 10–12 of the multivariate PSI values were combined to produce trends in the synchrony, because these combinations best represented changes in trends of the synchrony in response to gliches.

#### 4.5.3. Results of the analysis

**Figure 9.**
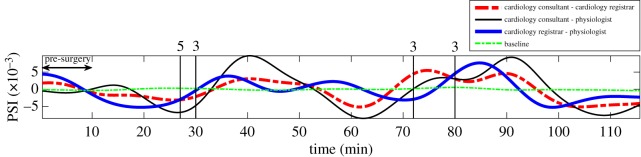
Trends in synchrony in the cardiologists’ and physiologist’s HRV data. Notation: baseline of the degree of synchrony (thin broken line), trends in the degree of synchrony between a cardio-consultant and a cardio-registrar (thick broken line), a cardio-consultant and a physiologist (thin solid line), and a cardio-registrar and a physiologist (thick solid line). The trends were estimated using the N-IPS algorithm, whereby 40 s sliding windows with 36 s overlap (4 s increment) were used in the PSI estimation. Two groups of glitches were recorded during the procedure: group 3 (distractions) and group 5 (equipment design). The recording was 116 min long.

Figure 9 shows trends in synchrony in HRV data during the recorded procedure, which exhibited cooperation in responses to glitches between the following pairs: (i) cardiology consultant–cardiology registrar, (ii) cardiology consultant–physiologist and (iii) cardiology registrar–physiologist. Glitches, which were recorded during the procedure included the following groups: (i) group 3—distractions–anything causing distraction from task (phone calls/bleeps, loud music, alarms), and (ii) group 5–equipment design—issues arising from equipment design that would not otherwise be corrected with training or maintenance (e.g. compatibility problems with different implants or wires, or equipment failure). Figure 9 shows that physiological responses of pairs of professionals responded to the recorded glitches with increased synchrony in HRV. Observe that the first glitch at the 27th min caused immediate negative deflections (increases) in trends of HRV synchrony between (i) the cardiology consultant and the physiologist (thin solid line) and (ii) the cardiology consultant and the cardiology registrar (thick broken line). Also, the third glitch at the 72nd min caused another immediate negative deflection (increase) in a trend of HRV synchrony between the cardiology registrar and the physiologist (thick solid line). These findings suggest these glitches were impeding the procedure and may affect procedural duration as well as outcome.

Simultaneous acquisition of HRV of team members involved allows us to objectively measure the involvement of each specialist during the case and observe their response to different interruptions and unexpected problems during the invasive cardiology procedures.

## 5. Conclusion

We have introduced a data-association measure which exhibits high frequency and time localization, termed ISC, for the analysis of nonlinear and non-stationary multivariate signals. This has been achieved through the combination of multivariate signal analysis using NA-MEMD and the generation of highly localized TF univariate and multivariate representations of the multivariate intrinsic oscillations using the STFT-based SST and MSST algorithms. Such a measure has enabled precise identification of physiological dependence in both frequency and time. The performance of the proposed ISC algorithm has been evaluated against other combinations of algorithms in the tasks of estimating synchrony in linear and nonlinear bivariate signals. The proposed ISC algorithm has been shown to outperform the other combinations of algorithms and to exhibit significantly higher average SNRs of TF representations of signal dependence. We have demonstrated an application of the ISC algorithm to the quantification of inter-channel dependence in respiratory and HRV signals. The proposed algorithm has exhibited highly localized synchrony in the respiratory and HRV signals among a subset of three bass singers of a choir during both a rehearsal and a performance. The proposed STFT-based ISC algorithm has been shown to outperform CWT-based ISC, which was built upon CWT-based SST and MSST, in the localization of synchronized frequencies in respiratory and HRV signals.

In addition to the ISC algorithm, we have proposed an extension of IPS, referred to as N-IPS, as a meaningful and straightforward-to-interpret data-association metric for trends in the level of cooperation. This is achieved by first employing the standard IPS to quantify intrinsic phase relationship between data channels, and then further decomposing time series of the multivariate degrees of phase synchrony into multiple scales, whereby certain intrinsic scales which contain physically meaningful and straightforward interpretation are then combined. This algorithm allows for empirical quantification of physically meaningful and straightforward-to-interpret trends in phase synchrony. Two applications of the N-IPS algorithm to the empirical quantification of physically meaningful trends in the level of cooperation through the empirical estimation of trends in synchrony in respiratory and HRV signals have been demonstrated. The N-IPS algorithm has effectively quantified physically meaningful increases and decreases in trends in levels of cooperation among subsets of soprano and bass singers of a choir during a 1 h evensong performance. It has also revealed significant increases in trends in levels of cooperation between pairs of cardiologists and physiologists when certain types of glitches occurred during a surgery. This is a first attempt to empirical quantification of intrinsic, physically meaningful and interpretable trends in the level of cooperation of long collaborative tasks.

Future work will focus on the development of Panorama-based SST and MSST algorithms in order to enhance the generation of TF representations [48]. The result of the surgical synchrony analysis is preliminary, and the N-IPS algorithm will be employed in the assessment of HRV changes in response to glitches within different surgical teams, to show how team familiarity has an impact on patient outcomes, i.e. complications, reintervention rate, length of surgical or endovascular procedures and length of post-operative hospitalization. A growing body of the literature suggests that shared knowledge and understanding of each other’s roles and objectives during the invasive and high-risk procedures in turn facilitates team cooperation and coordination. For a team to function efficiently, its members should share a ‘mental model’ of the team’s tasks, objectives, means and environment [49–51]. We are hoping that our prospective research will help us to facilitate a better training programme across UK hospitals in order to deliver better care to the patients.

## Figures and Tables

**Figure 10.**
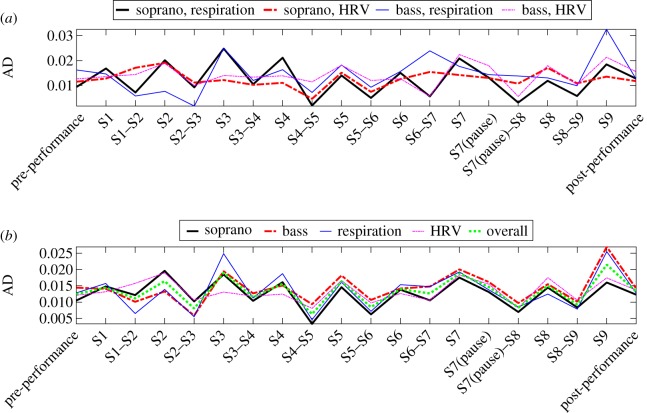
Areal densities of trends in synchrony. (*a*) Areal density of trend in synchrony in the sopranos’ respiratory signals (solid thick line), the sopranos’ HRV signals (broken thick line), the basses’ respiratory signals (solid thin line) and the basses’ HRV signals (broken thin line). (*b*) Average areal density of trends in synchrony in both signals of the sopranos (solid thick line), both signals of the basses (broken thick line), the respiratory signals of both voices (solid thin line), the HRV signals of both voices (broken thin line) and all the signals (dotted thick line).

## Data accessibility

Data available from the Dryad Digital Repository: http://dx.doi.org/10.5061/dryad.80cv0 [52]. It can also be accessed from http://www.commsp.ee.ic.ac.uk/~mandic/research/emd.htm.

## Appendix A

**A.1. Noise-assisted multivariate empirical mode decomposition**

It has been shown in [53,54] that EMD acts essentially as a dyadic filter when decomposing WGN, enforcing the dyadic filterbank structure onto IMFs—Fourier spectra of the IMFs are all identical and cover the same area on a semi-logarithmic period scale. Therefore, the aim of simultaneously decomposing added WGN channels and the input signal using noise-assisted MEMD (NA-MEMD) is to enforce the dyadic filterbank structure within the IMFs by using WGN as a decomposition reference and to reduce mode mixing. *While this may hinder the data-driven operation of MEMD, it is essential in multichannel operations where the requirement is to compare IMFs with similar centre frequencies and bandwidths in order to preserve the physical meaning of the analysis.* It should also be noted that the most important aspect of an MEMD approach, via either standard MEMD or NA-MEMD, is that even if mode mixing occurs it is likely to happen simultaneously in every channel. In this way, multicomponent comparisons at the IMF level are matched and physically meaningful. It was also suggested in [8] that the number of WGN channels should be as large as possible—computation time permitting—in order to reduce the degree of overlap between IMF spectra, that is, impose a more pronounced dyadic filterbank structure within the IMFs. See algorithm 3 for details of the NA-MEMD algorithm.

**Algorithm 3:** Noise-assisted multivariate empirical mode decomposition.

**Input:** A multivariate signal **y**(*t*), **y**(*t*)=[*y*_1_(*t*),*y*_2_(*t*),…,*y*_*N*_(*t*)]^*T*^.

(i) Construct a P-variate WGN signal **z**(*t*), **z**(*t*)=[*z*_1_(*t*),*z*_2_(*t*),…,*z*_*P*_(*t*)]^*T*^.
(ii) Treat both signals **y**(*t*) and **z**(*t*) as a single (N + P)-variate signal **x**(*t*), **x**(*t*)=[*y*_1_(*t*),*y*_2_(*t*),…,*y*_*N*_(*t*),*z*_1_(*t*),*z*_2_(*t*),…,*z*_*P*_(*t*)]^*T*^.
(iii) Let multivariate proto-IMF $\tilde{\text{x}}(t)=\text{x}(t)$, and *R*=*N*+*P*.
(iv) Choose a weighting scheme based on a suitable pointset for sampling on a (*R*)-hypersphere, ${\text{w}}_{q}={\left\{w_{\left\{q,1\right\}},w_{\left\{q,2\right\}},\ldots ,w_{\left\{q,R\right\}}\right\}}_{q=1}^{Q}$.
(v) Calculate a projection, denoted by $\Upsilon {\left(\tilde{\text{x}}(t)\right)}_{{\text{w}}_{q}}$, of the proto-IMF $\tilde{\text{x}}(t)$ along the direction vector **w**_*q*_, for all *q* (the whole set of direction vectors), giving ${\left\{\Upsilon (\tilde{\text{x}}{\left(t)\right)}_{{\text{w}}_{q}}\right\}}_{q=1}^{Q}$ as the set of projections.
(vi) Find the j=1,2,…,J time instants $\left\{t_{j,\text{min}}^{{\text{w}}_{q}}\right\}$ and $\left\{t_{j,\text{max}}^{{\text{w}}_{q}}\right\}$ corresponding, respectively, to the minima and maxima of the set of projected signals ${\left\{\Upsilon (\tilde{\text{x}}{\left(t)\right)}_{{\text{w}}_{q}}\right\}}_{q=1}^{Q}$.
(vii) Interpolate $\tilde{\text{x}}(t_{j,\text{min}}^{{\text{w}}_{q}})$ and $\tilde{\text{x}}(t_{j,\text{max}}^{{\text{w}}_{q}})$ to obtain, respectively, multivariate minima and maxima envelope curves ${\left\{{\text{e}}_{\text{min}}^{{\text{w}}_{q}}(t)\right\}}_{q=1}^{Q}$ and ${\left\{{\text{e}}_{\text{max}}^{{\text{w}}_{q}}(t)\right\}}_{q=1}^{Q}$.
(viii) For a set of *Q* direction vectors, the mean **m**(*t*) of the envelope curves is calculated as:
  A 1 $$\text{m}(t)=\frac{1}{2Q}\sum_{q=1}^{Q}{\text{e}}_{\text{min}}^{{\text{w}}_{q}}(t)+{\text{e}}_{\text{max}}^{{\text{w}}_{q}}(t).$$
(ix) Extract the detail **d**(*t*) using $\text{d}(t)=\tilde{\text{x}}(t)-\text{m}(t)$.
(x) If the detail **d**(*t*) fulfils the stoppage criterion for a multivariate IMF (the difference between two consecutive sifting results is below a certain threshold), **d**(*t*) becomes a multivariate IMF, **c**_*k*_(*t*), and $\tilde{\text{x}}(t)=\tilde{\text{x}}(t)-{\text{c}}_{k}(t)$. Otherwise, $\tilde{\text{x}}(t)=\text{d}(t)$.
(xi) Repeat steps (v)–(x) until $\tilde{\text{x}}(t)$ is a multivariate monotonic residue or trend.

**A.2. Short-time Fourier transform-based synchrosqueezing transform**

The synchrosqueezing transform (SST) [27] is a post-processing technique originally applied to the CWT in order to generate highly localized TF representations of nonlinear and non-stationary signals. It has been extended to the STFT setting in [28], and is referred to as STFT-based SST (FSST). It performs STFT, instead of CWT, on a given univariate signal *x*(*t*) to obtain STFT coefficients *U*(*η*,*t*), given by

A 2 $$U(\eta ,t)=\int_{ℜ}^{\;}x(\tau )g(\tau -t)\;e^{-2i\pi \eta (\tau -t)}\;d\tau ,$$

where *g* is a sliding window and *τ* is the time lag. The STFT coefficients, *U*(*η*,*t*), are next relocated to the instantaneous frequency, *F*, according to the map (*η*,*t*)↦(*F*(*η*,*t*)), where *F* is defined by

A 3 $$F(\eta ,t)=\frac{1}{2\pi }\partial_{t}\;\text{arg}\;U(\eta ,t)=Re\left(\frac{1}{2i\pi }\frac{\partial_{t}U(\eta ,t)}{U(\eta ,t)}\right).$$

The STFT-based SST coefficient is then given by

A 4 $$T(\;f,t)=\frac{1}{g(0)}\int_{ℜ}^{\;}U(\eta ,t)\delta (\;f-F(\eta ,t))\;d\eta .$$

A summary of the FSST is outlined in algorithm 4.

**Algorithm 4:** Short-time Fourier transform-based synchrosqueezing transform.

(i) Given a univariate signal *x*(*t*), perform STFT on the signal in order to obtain the STFT coefficients *U*(*η*,*t*), given by equation A.2.
(ii) Estimate the instantaneous frequency *F*(*η*,*t*) of the STFT coefficients *U*(*η*,*t*), given by equation A.3.
(iii) Combine the STFT coefficients in order to obtain the STFT-based SST coefficients *T*( *f*,*t*), given by equation A.4.

**A.3. Short-time Fourier transform-based multivariate synchrosqueezing transform**

The multivariate synchrosqueezing transform (MSST) [29] is an extension of the CWT-based SST to multivariate cases. It was proposed in order to identify oscillations common to multiple data channels. It first partitions the TF domain into *M* frequency bands {*f*_m_}_*m*=1,2,…,*M*_. This makes it possible to identify a set of matched monocomponent signals from a given multivariate signal. The instantaneous amplitudes and frequencies present within those frequency bands can then be determined. The implementation of F-MSST is straightforward; FSST is employed instead of SST to obtain SST coefficients of each channel.

For an N-variate signal **x**(*t*) with the corresponding SST coefficients of each channel *n*, *T*_*n*_( *f*,*t*) obtained using FSST, and a set of frequency bands {*f*_m_}_*m*=1,2,…,*M*_, the instantaneous frequency *F*( *f*_m_,*t*) for each channel *n* and frequency band *m* is given by

A 5 $$F(\;f_{m},t)=\frac{\sum_{f\in f_{m}}^{\;}|T_{n}(\;f,t){|}^{2}f}{\sum_{f\in f_{m}}^{\;}|T_{n}(\;f,t){|}^{2}}$$

and the instantaneous amplitude *A*( *f*_m_,*t*) for each channel and frequency band is given by

A 6 $$A(\;f_{m},t)=\sqrt{\sum_{f\in f_{m}}^{\;}|T_{n}(\;f,t){|}^{2}}.$$

To estimate the multivariate instantaneous frequency **F**( *f*_m_,*t*) for a given frequency band *m*, the instantaneous frequencies across the *N* channels are then combined using

A 7 $$\text{F}(\;f_{m},t)=\frac{\sum_{n=1}^{N}(A{\left(\;f_{m},t)\right)}^{2}F(\;f_{m},t)}{\sum_{n=1}^{N}(A{\left(\;f_{m},t)\right)}^{2}},$$

while the multivariate instantaneous amplitude **A**( *f*_m_,*t*) for each frequency band is given by

A 8 $$\text{A}(\;f_{m},t)=\sqrt{\sum_{n=1}^{N}(A{\left(\;f_{m},t)\right)}^{2}}.$$

The multivariate STFT-based synchrosqueezing transform coefficient **T**( *f*_m_,*t*) for each frequency band *m* is given by

A 9 $$\text{T}(\;f_{m},t)=\text{A}(\;f_{m},t)\delta (\;f-\text{F}(\;f_{m},t)),$$

where *δ*(⋅) is the Dirac delta function. The multivariate STFT-based SSTs for all frequency bands are then given by

A 10 $$\text{T}(\;f,t)=\text{T}(\;f_{m},t){|}_{m=1,2,\ldots ,M}.$$

A summary of the F-MSST is outlined in algorithm 5.

**Algorithm 5:** STFT-based multivariate synchrosqueezing transform.

(i) Given an N-variate signal **x**(*t*), apply FSST channel-wise in order to obtain the univariate FSST coefficients *T*_*n*_( *f*,*t*).
(ii) Determine a set of partitions along the frequency axis for the TF domain, and calculate the instantaneous frequency *F*( *f*_m_,*t*) and amplitude *A*( *f*_m_,*t*) for each frequency band *m* and channel *n*, given by equations A.5 and A.6, respectively.
(iii) Calculate the multivariate instantaneous frequency **F**( *f*_m_,*t*) and amplitude **A**( *f*_m_,*t*) for each channel *m*, using equations A.7 and A.8, respectively.
(iv) Determine the multivariate STFT-based synchrosqueezing coefficients **T**( *f*,*t*), using equations A.9 and A.10.

**A.4. Areal density**

Trends in synchrony obtained using the proposed N-IPS algorithm offer a meaningful and straightforward interpretation in signal dependence. This can also be further simplified by calculating the areal density (AD)—an area between a trend in synchrony and a corresponding baseline per unit of time. Figure 10*a* shows areal densities of trends in synchrony in the respiratory and HRV signals of the soprano and bass singers considered in §4.4. Observe that the areal densities increased during the songs (S_*x*_, where *x* represents the song number) and decreased between two consecutive songs (S_*x*_–S_*y*_, where *x* and *y* represent the two consecutive song numbers), except for only two circumstances: (i) during Songs 1 and 2 (S1–S2), where areal densities of the trend in synchrony in the HRV signals of the soprano and bass singers remained relatively high; and (ii) during Songs 6 and 7 (S6–S7), where areal densities of the trends in synchrony in the sopranos’ HRV signals and the basses’ respiratory signals increased from the previous song (Song 6, S6). Note that ‘S7 (pause)’ represents the later period of Song 7 where several pauses occurred, and consequently the trends and the areal densities of the trends in synchrony decreased. For rigour, we also calculated average areal densities of trends in synchrony in both signals of the sopranos and basses, as shown in figure 10*b*. The average areal densities exhibited distinct increases during the songs and decreases between the songs.
